# Reframing livestock antimicrobial use as a global public good

**DOI:** 10.1016/j.onehlt.2026.101349

**Published:** 2026-02-02

**Authors:** Alejandro Acosta, Francesco Nicolli, Rachel Dalton, Antonio Valcarce, Emmanuel Kabali, Alejandro Dorado Garcia, Carmen Bullon, Wondmagegn Tirkaso, Junxia Song

**Affiliations:** a Animal Production and Health Division, Food and Agriculture Organization of the United Nations (FAO), Rome, Italy; b Development Law Service, Food and Agriculture Organization of the United Nations (FAO), Rome, Italy

**Keywords:** Livestock, Global Public Goods, Antimicrobial Effectiveness, Antimicrobial Use, Antimicrobial Resistance, Antimicrobial Stewardship, One Health

## Abstract

Global antimicrobial use in livestock is projected to rise over coming decades, accelerating antimicrobial resistance and eroding antimicrobial effectiveness. Although stewardship efforts have expanded, they remain largely national and voluntary, even as resistance dynamics propagate across borders. This paper reframes antimicrobial effectiveness in livestock systems as a global public good, defined by non-rivalry, non-excludability, and transboundary spillovers. Under this framing, persistent overuse reflects governance and market failures that misalign local production incentives with global welfare losses. Drawing on global public good theory within a One Health perspective, the paper develops an integrated stewardship framework that clarifies why aggregating national actions underdelivers. The framework links four mutually reinforcing pillars: international governance and accountability, incentive-compatible economic instruments, sustainable and equitable financing, and farm-level adoption enablers. Together, these pillars translate shared global objectives into national stewardship while preserving access for animal health needs, thereby supporting livestock productivity, food security, and public health.

## 1 Introduction

By 2040, antimicrobial use (AMU) in livestock is projected to rise by nearly 30% [1]. This trend is not driven solely by technical constraints, but by governance and incentive structures that allow one of humanity's most valuable health resources to be eroded by short-term private gains [10], [18]. Antimicrobial overuse in livestock accelerates antimicrobial resistance (AMR) at a pace that outstrips mitigation efforts, with consequences that extend well beyond farms [2]. Resistant pathogens and resistance genes move across borders, sectors, and species, threatening both animal and human health [13], [23].

What is at stake is not the physical availability of antimicrobials, but their effectiveness, understood as the ability of these drugs to prevent or treat infections over time. Antimicrobial effectiveness is inherently shared across borders. When one country reduces unnecessary AMU, others benefit. When resistance emerges in one setting, treatment options can deteriorate elsewhere. As a result, the benefits of stewardship are global and long term, while the economic gains from overuse remain immediate and local [10]. This misalignment between private incentives and social costs contributes to AMU that exceeds socially optimal levels.

Political recognition of this challenge has increased, from the 2022 Muscat Ministerial Manifesto to the 2024 United Nations General Assembly Declaration and the 2024 Jeddah Commitments [7], [9], [21]. Yet policy responses remain fragmented and largely voluntary. Technical measures such as improved veterinary oversight, enhanced surveillance, and better husbandry practices are essential, but insufficient when implemented in isolation [6]. Without enforceable governance and better-aligned incentives, national stewardship plans are unlikely to deliver sustained reductions in AMU at scale.

From an economic perspective, the inability to confine antimicrobial effectiveness within national boundaries implies that even well-designed domestic policies may fall short. Reductions in AMU generate benefits beyond national borders, while resistance arising in one location can undermine treatment efficacy elsewhere through trade, travel, and environmental pathways. This cross-border interdependence points to a collective action problem that cannot be resolved through national efforts alone and highlights the need for coordinated international action under a One Health approach.

Against this background, framing antimicrobial effectiveness as a global public good (GPG) offers a useful economic lens for understanding why existing stewardship efforts remain insufficient and for structuring more coherent responses. Rather than assuming a lack of concern among producers or governments, this perspective highlights the structural gap between local decision making and global consequences. Building on this framing, the paper examines how governance arrangements and economic instruments might help better align incentives across farms, value chains, national authorities, and international coordination, with the aim of slowing the erosion of antimicrobial effectiveness and safeguarding livestock productivity, food security, and public health.

## 2 Challenges with existing approaches

Current antimicrobial stewardship efforts in livestock are predominantly framed at the national or sectoral level. While such initiatives can generate measurable improvements, their effectiveness is inherently limited when the underlying challenge is global in scope and shaped by collective action problems [10], [18].

A central weakness is fragmented governance. National action plans are often voluntary, unevenly enforced within and across countries, and rarely designed with mechanisms to account for the cross-border spillovers of national decisions [6], [17]. As a result, a country may adopt strict regulations on AMU in livestock production yet still face rising resistance levels if neighbouring or trading partners maintain high-use practices. Resistant bacteria and resistance genes can spread through trade in live animals, food products, and feed, as well as via environmental pathways such as water systems [22].

These governance challenges are further compounded in contexts where antimicrobials are accessed through informal or unauthorized supply chains. In such settings, stewardship policies that rely on regulated distribution channels and formal compliance mechanisms face inherent limitations, as enforcement capacity is weakened and use decisions occur outside official oversight. Recent work highlights that widespread over-the-counter access to antibiotics in many low- and middle-income countries reflects broader health system constraints rather than isolated regulatory failure, underscoring the need for stewardship approaches that are compatible with existing supply realities while progressively strengthening formal control mechanisms [15].

Economic incentives further weaken stewardship efforts. Livestock producers often derive immediate private benefits from AMU, including reduced disease risk, higher productivity, and more predictable output. By contrast, the costs associated with AMR, such as declining treatment efficacy, increased morbidity, and higher veterinary and medical expenditures, are diffuse, delayed, and largely absent from market prices [10]. In the absence of corrective policies, these incentives encourage levels of AMU that exceed what would be socially optimal.

Structural constraints also limit the effectiveness of technical interventions, particularly in low- and middle-income countries. Limited infrastructure, inadequate veterinary services, and restricted access to diagnostics or vaccines often prevent improved practices from reaching scale [8], [24]. Even market-based instruments such as certification schemes or residue monitoring can reinforce inequities if compliance costs fall disproportionately on smallholders or exporters without accompanying support [20], [25].

Taken together, these limitations indicate that current stewardship approaches, while necessary, are insufficient when pursued primarily through voluntary or domestic measures. Addressing AMR in livestock therefore requires moving beyond the aggregation of national action plans toward governance arrangements that can coordinate action across borders, align incentives with shared outcomes, and support implementation according to capacity and impact.

## 3 Reconceptualizing antimicrobial use through a global public good lens

The persistent overuse of antimicrobials in livestock reflects the logic of a shared resource whose benefits are immediate and private, while the costs are delayed and widely dispersed. Viewing antimicrobial effectiveness through a GPG lens helps explain why stewardship efforts that rely primarily on national action and voluntary compliance struggle to deliver sustained reductions in use [3], [11].

Antimicrobial effectiveness exhibits three defining economic properties that underpin the collective action problem. It is non-rival in the sense that prudent use by one actor does not reduce its availability to others. It is also effectively non-excludable at the global level, since preserved effectiveness cannot be confined within national borders and resistance emerging in one setting can undermine treatment options elsewhere. Finally, it generates transboundary spillovers, as trade, travel, and environmental pathways facilitate the spread of resistant organisms and genes, linking local use decisions to global outcomes [22]. In the absence of coordinated governance, these characteristics create strong incentives for overuse [10], [18].

At the producer level, AMU generates tangible private benefits, including reduced mortality, improved feed conversion, and more predictable output. By contrast, the broader social costs associated with AMR accrue slowly and often outside the sector or country where use occurs. Because these costs are not reflected in production decisions, AMU tends to exceed socially optimal levels [[4], [14]].

Economic instruments offer one avenue to address this imbalance. Tradable permit systems can establish enforceable caps on AMU while allowing flexibility through exchange among users [18]. Taxes on veterinary antimicrobials can discourage non-essential use and generate resources for investment in preventive measures and alternatives [16]. To be effective, such instruments must be paired with measures that reduce the cost and risk of adopting vaccines, diagnostics, biosecurity, and improved husbandry practices [22].

The global impact of AMU also depends on how individual actions aggregate. In the context of AMR, all countries contribute to the global resistance pool, but those with large livestock sectors or high AMU intensity exert disproportionate influence [19]. Strong veterinary services and stewardship systems can mitigate these effects, while institutional weaknesses can amplify risks, even in smaller producing countries, if resistant pathogens spread through trade or live animal movements.

Trade plays a dual role in this context. On one hand, it facilitates the transmission of resistant organisms across borders. On the other, it can create incentives for improved stewardship when access to high-value markets depends on compliance with standards set by Codex Alimentarius and the World Organization for Animal Health (WOAH). While some exporters have successfully aligned with these standards, others have faced trade disruptions linked to regulatory gaps [5]. For low- and middle-income countries, meeting higher requirements often requires targeted investment support to avoid exacerbating existing inequities [6], [12].

Taken together, non-rivalry, non-excludability, and transboundary spillovers create a classic commons problem. Addressing it requires institutions capable of translating shared global objectives into credible incentives at national and sectoral levels, embedding stewardship within trade and market structures, and supporting monitoring systems that enable mutual accountability. Without such arrangements, antimicrobial effectiveness will continue to erode, with consequences that are both irreversible and widely shared.

## 4 Toward a global framework for antimicrobial stewardship

**Fig. 1:**
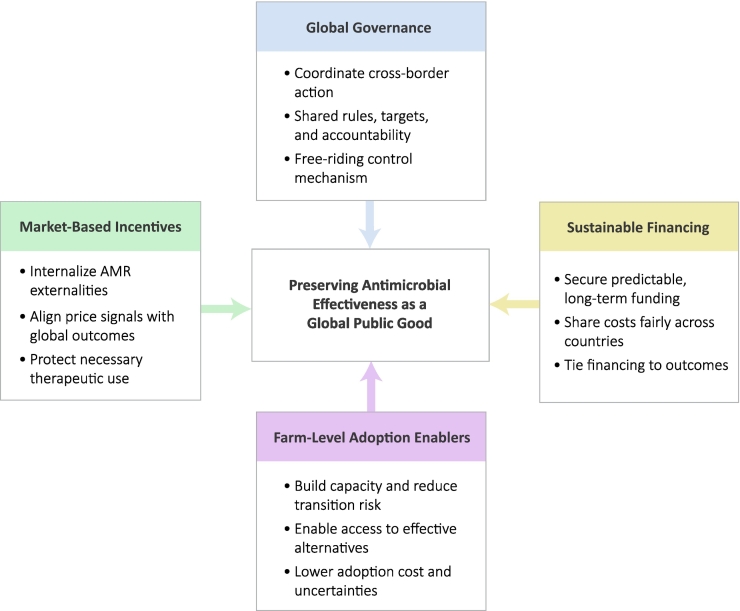
Integrated framework for preserving antimicrobial effectiveness as a global public good in livestock systems. The framework places antimicrobial effectiveness at the center and illustrates how stewardship outcomes emerge from the interaction of four mutually reinforcing pillars: international governance, economic incentives, sustainable financing, and farm-level adoption enablers.

Recognizing antimicrobial effectiveness as a GPG provides a basis for rethinking the policy and institutional arrangements required to address AMR. This section synthesizes the preceding analysis into an integrated framework for antimicrobial stewardship, summarized in Fig. 1, which places antimicrobial effectiveness at the center and highlights how outcomes depend on the interaction of international governance, economic incentives, sustainable financing, and farm-level adoption. Rather than prescribing specific instruments, the framework illustrates how these elements must align to support sustained reductions in AMU while preserving access for animal health needs.

Progress has been made through the Quadripartite collaboration among FAO, WHO, WOAH, and UNEP, which has strengthened technical guidance and coordination through mechanisms such as the AMR Multi-Stakeholder Partnership Platform and the Global Leaders Group on AMR. However, these arrangements remain largely advisory and lack the authority needed to ensure consistent implementation or accountability across countries [6], [17]. Strengthening the Quadripartite Joint Secretariat through clearer mandates, predictable operational resources, and enhanced capacity to support national implementation could improve coherence across sectors. Recent political recognition of its role under the One Health framework in the 2024 United Nations General Assembly Political Declaration provides an opportunity to further institutionalize this function.

Independent scientific input is also essential for credible decision making. The Independent Panel on Evidence for Action against AMR (IPEA), endorsed in the 2024 Declaration and outlined in the Quadripartite AMR Roadmap, is intended to provide integrated assessments and policy-relevant guidance across sectors. For this body to be effective, it will require institutional independence, adequate resourcing, and a clearly defined role in informing target setting, monitoring progress, and promoting learning across countries. Over time, more formal international arrangements could help clarify shared responsibilities and support sustained collective action.

Governance reforms alone are unlikely to correct the market failures that drive antimicrobial overuse. Producers often face immediate private benefits from AMU through reduced disease risk and more predictable output, while the broader costs associated with resistance remain diffuse and delayed [10], [18]. Economic instruments can help internalize these costs. Approaches such as tradable allowance systems or targeted taxation may discourage unnecessary use while preserving access for therapeutic purposes, provided they are designed with sufficient flexibility to respond to animal health needs [16], [18]. These instruments are most effective when combined with measures that reduce the cost and risk of adopting preventive practices, including vaccination, diagnostics, improved biosecurity, and husbandry improvements [22].

Financing is a critical link between policy ambition and implementation. Many countries, particularly in low- and middle-income groups, lack dedicated budget lines for AMR and continue to rely heavily on short-term external funding [6]. Integrating antimicrobial stewardship into national budgetary processes and public investment planning can improve sustainability and strengthen national ownership. At the international level, blended finance, concessional lending, and results-based mechanisms may help mobilize resources for investments that reduce dependence on antimicrobials, including animal health infrastructure and preventive technologies.

Beyond overall resource mobilization, financing arrangements should reflect equitable burden-sharing given the cross-border benefits of stewardship. Countries that deliver substantial global benefits through reductions in AMU may warrant co-financing, particularly where domestic fiscal space is limited. Linking financial support to measurable outcomes, such as verified reductions in use or strengthened surveillance capacity, can reinforce accountability. Global monitoring platforms, including the Global Antimicrobial Resistance and Use Surveillance System (GLASS), Global Database on Animal Antimicrobial Use (ANIMUSE), International FAO Antimicrobial Resistance Monitoring System (InFARM), and the Global Integrated System for Surveillance of Antimicrobial Resistance and Use (GISSA), provide a basis for tracking progress and supporting mutual confidence.

Ultimately, the effectiveness of stewardship efforts depends on decisions made at the farm level. While price signals matter, they are insufficient if producers lack access to veterinary services, diagnostics, credit, or reliable information. Capacity-building initiatives such as Farmer Field Schools, veterinary outreach, and peer learning can support behavioural change by reducing uncertainty and strengthening confidence in preventive practices [8], [24], and can reinforce appropriate sourcing by increasing awareness of veterinary oversight and the importance of obtaining antimicrobials through authorized supply channels [15].

Actors along the value chain also play an important role. Processors and retailers can reinforce stewardship by integrating AMU criteria into procurement standards, offering price premiums, or establishing longer-term contractual arrangements. Public policy can complement these efforts through co-financing, risk-sharing mechanisms, and targeted support for investments in housing, biosecurity, and animal health. Without such enabling conditions, resource-constrained producers are likely to remain locked into production systems that depend heavily on AMU.

## 5 Conclusion

Preserving antimicrobial effectiveness is ultimately a test of whether the international community can act collectively to safeguard a resource whose value depends on responsible use and effective governance to limit the gradual and often invisible spread of resistance. In livestock systems, this challenge is shaped not only by technical constraints but by structural misalignments between local decision making and global consequences. Antimicrobial effectiveness exhibits the defining characteristics of a GPG, namely non-rivalry, non-excludability, and transboundary spillovers, which makes it particularly vulnerable to overuse when stewardship relies primarily on voluntary or nationally bounded approaches.

Many of the elements required to address this challenge already exist. Technical guidance, surveillance systems, market instruments, and preventive practices are well established, and recent political commitments signal growing recognition of the problem. Yet these elements remain insufficiently integrated within a governance architecture capable of aligning incentives, mobilizing sustained financing, and supporting implementation across countries and production systems. The central gap is therefore institutional rather than technological.

By reframing antimicrobial effectiveness as a GPG, this perspective offers an economic rationale for strengthening international cooperation and for linking national stewardship efforts more explicitly to shared global outcomes. Rather than prescribing specific policy instruments, the analysis highlights the importance of coordinated governance, incentive-compatible economic tools, predictable financing, and farm-level capacity as mutually reinforcing components of effective stewardship. Advancing in this direction would help move the global response beyond fragmented action toward a more coherent and equitable approach under a One Health framework.

Whether antimicrobial effectiveness can be preserved over the long term will depend on the ability of countries and international institutions to translate shared recognition into credible collective action. Failure to do so risks continued erosion of a resource that underpins livestock productivity, food security, and public health worldwide.

## CRediT authorship contribution statement

**Alejandro Acosta:** Writing – original draft, Conceptualization. **Francesco Nicolli:** Writing – original draft, Conceptualization. **Rachel Dalton:** Writing – original draft. **Antonio Valcarce:** Writing – review & editing. **Emmanuel Kabali:** Writing – original draft. **Alejandro Dorado Garcia:** Writing – original draft. **Carmen Bullon:** Writing – original draft. **Wondmagegn Tirkaso:** Visualization, Writing – review & editing, Investigation. **Junxia Song:** Writing – original draft.

## Declaration of competing interest

The authors declare no competing interests.

## Data Availability

No data was used for the research described in the article.
